# Development and Validation of a Stability-Indicating Assay of Etofenamate by RP-HPLC and Characterization of Degradation Products

**DOI:** 10.3797/scipharm.1305-19

**Published:** 2013-07-22

**Authors:** Ramalingam Peraman, Devanna Nayakanti, Hari Hara Theja Dugga, Sudhakara Kodikonda

**Affiliations:** 1 Analytical Research Laboratory, Raghavendra Institute of Pharmaceutical Education and Research (RIPER), Anantapur (JNTUA), Andhra Pradesh – 515721, India.; 2 Jawarharlal Nehru Technological University Anantapur, Andhra Pradesh – 515001, India.

**Keywords:** Etofenamate, Stability, RP-HPLC, NMR, Mass spectral studies

## Abstract

A validated stability-indicating RP-HPLC method for etofenamate (ETF) was developed by separating its degradation products on a C18 (250 mm × 4.6 mm 5 μm) Qualisil BDS column using a phosphate buffer (pH-adjusted to 6.0 with orthophosphoric acid) and methanol in the ratio of 20:80 % *v/v* as the mobile phase at a flow rate of 1.0 mL/min. The column effluents were monitored by a photodiode array detector set at 286 nm. The method was validated in terms of specificity, linearity, accuracy, precision, detection limit, quantification limit, and robustness. Forced degradation of etofenamate was carried out under acidic, basic, thermal, photo, and peroxide conditions and the major degradation products of acidic and basic degradation were isolated and characterized by ^1^H-NMR, ^13^C-NMR, and mass spectral studies. The mass balance of the method varied between 92–99%.

## Introduction

Etofenamate (ETF) is chemically 2-{[3-(trifluoromethyl)phenyl]amino}benzoic acid 2-(2-hydroxyethoxy)ethyl ester, which exists as viscous a liquid and is used as an analgesic, antirheumatic, antipyretic, and anti-inflammatory [1]. It works by blocking an enzyme in the body known as cyclooxygenase (COX). Etofenamate is marketed in the form of injection and topical gel for the treatment of various conditions like arthritis, muscular rheumatism, muscle pain, periarthritis of the shoulder, joint pain, lower back pain (lumbago), sciatica (ischialgia), sports injuries, and tendonitis [2].

An important factor that affects the safety of a drug’s use is its chemical stability. The efficacy is attributed to the ester and ether linkages which are very susceptible to chemical degradation. During long-term storage, various stress factors like moisture, temperature, and light may induce degradation of the active ingredients. Thus, the formed degradants need to be separated in a quantification procedure by an appropriate analytical method. Literature reveals that few bioanalytical [3, 4], high-performance liquid chromatographic [5], or gas chromatographic [6] methods have been reported for ETF. Although various methods are reported for the determination of etofenamate, they are selective only for the estimation of ETF in biological fluids. As to date, no stability studies on ETF have been reported, therefore the present work was designed to develop a sensitive, precise, and selective stability-indicating RP-HPLC method for the estimation of ETF in dosage forms and to isolate and characterize the major degradants by spectral studies. The method is validated as per the guidelines of the International Conference on Harmonization (ICH) [7]. The stress tests are employed to establish the effect of temperature, humidity, light, oxidizing agent, and pH on the formation of degradants in ETF dosage forms [8].

## Experimental

### Materials and Reagents

All reagents and solvents were of HPLC grade procured from Merck, India. Etofenamate (99.66%) was supplied by UQUIFEA, Barcelona, Spain as a gift sample.

### Instrumentation

The HPLC system (Agilent LC Model-1200 with Ez Chromelite Software) equipped with a C_18_ (Qualisil BDS, 250 × 4.6 mm, 5μ) column, DAD detectors, and a Rheodyne manual injector was used. The LABINDIA-3000^+^ UV-Visible double beam spectrophotometer with a fixed slit width of 1nm- and 1cm-matched quartz cells was used for all the spectral measurements. The pH measurements were carried out with the Eutech pH Tutor, a pH meter equipped with a combined glass-calomel electrode, which was calibrated using standard buffer solutions of pH 4.0 and 7.0. The Analogix intelli Flash LC System with a gradient pump and VWD-UV detector were used for the isolation and purification of degradants.

### Solution State Stability

4 mL of primary stock solution was withdrawn into a 10 mL volumetric flask and made up to 10 mL with the mobile phase. Periodically, 1 mL of the solution was diluted to 10 mL with the mobile phase and injected into the column against the blank.

### Forced Degradation Studies of Etofenamate (ETF)

Forced degradation of ETF was performed under neutral, acid, alkaline, oxidative, thermal, and photolytic stress conditions [8]. Stress was induced at a concentration of 1000 μg/mL periodically, aliquots were diluted with the mobile phase to 40 μg/mL, neutralized to pH 5–7, and injected under the optimized chromatographic conditions with an appropriate blank for the control and stress. Blank solutions for each hydrolytic stress study were prepared simultaneously along with stock solutions.

### Preparation of Stock Solution for Stress Studies

An amount of 100mg of the drug substance was accurately weighed, transferred into a 100 mL volumetric flask, dissolved completely in methanol, and the volume was made up to get 1000 μg/mL. Stress solutions were prepared by diluting 4 mL of the above solution to 10 mL using HCl (0.1 – 1N), NaOH (0.01 – 1N), and H_2_O_2_ (0.3 – 3%), respectively, for acid hydrolysis, base hydrolysis, and oxidative stress. Thermal degradation was carried out for the liquid state by heating the sample as a thin layer in a hot air oven at 70°C. Photodegradation was carried out under hot sunlight.

### Hydrolysis

Stress solutions of 400 μg/mL were prepared in 0.01N NaOH (basic), 0.1N HCl (acidic), and water (neutral) at room temperature. One-mL of the sample was withdrawn at different time points and made to 10 mL with the mobile phase (40 μg/mL). The sample from acid hydrolysis was neutralized with 10% NaOH and the sample from base hydrolysis was neutralized with 10% HCl before injection into the column.

### Oxidation

Four-mL of stock solution was withdrawn into a 10 mL volumetric flask and made to 10 mL with 0.3% H_2_O_2_. Periodically, 1 mL of stress solution was diluted to 10 mL with the mobile phase and injected into the optimized conditions against the control and stress blank.

### Thermal Degradation

It was performed on the preheated sample as a thin layer in the petridish at 70°C. At various time intervals, 10 mg of the heated samples were weighed, dissolved, and suitably diluted with the mobile phase to a concentration of 40 μg/mL and injected into the system.

### Photodegradation

Photodegradation studies were conducted by exposing the liquid sample to hot sunlight for a period of 72h. Every 4h, the stock solution was prepared, diluted suitably with the mobile phase to a concentration of 40μg/mL and injected into the system.

### Synthesis, Purification, and Characterization of Acidic and Alkali Degradation Products

#### Acid Hydrolysis

An amount of 0.003 mol of etofenamate was dissolved in 10 mL of methanol. To this, 5 mL of 50% HCl was added and refluxed for 1h at 60°C. The reaction mixture was neutralized with alkali to pH 7 and kept in a refrigerator overnight. The formed cloudy precipitate was then isolated and purified by flash chromatography using a silica gel column.

#### Base Hydrolysis

An amount of 0.003 mol of etofenamate was dissolved in 10 mL of methanol and 5mL of 10% NaOH was added and refluxed for 2h at 60°C. The formed degraded product was precipitated by acidification with 1N HCl. The crude product obtained was recrystallized and purified on a silica gel column by flash chromatography.

#### Purification

The products were subjected to purification by flash chromatography using silica gel as the column material and a mixture of methanol and ethyl acetate (from 5% to 50% methanol) as the mobile phase in gradient mode. VWD-UV detection (using the software Analogix IF 280 V 5.10) was carried out for the collection of fractions. At the end of elution analysis, the fractions were combined based on the UV characteristics and TLC and evaporated in a vacuum dryer. Products (D1 and D2) were isolated and characterized by ^1^H-NMR, ^13^C-NMR, and mass spectral studies. These products were spiked with ETF and analyzed in optimized conditions to identify the degradants in the stress studies.

Acid degradant (D2): 2-hydroxyethyl 2-{[3-(trifluoromethyl)phenyl]amino}benzoate, Mol. formula: C_16_H_14_F_3_NO_3_, MW: 325, Yellow solid, UV max: 282 nm, ^1^H-NMR (CDCl_3_, δ ppm): 3.2 (2H, t, CH_2_), 3.4 (2H, t, CH_2_), 4.0 (1H, s, OH), 5.12–6.3 (8H, cpx, J = 13–15 Hz, Ar-H), 7.13 (1H, s, NH). ^13^C-NMR (CDCl_3_, δ ppm): 32.1, 43.4, 113.4, 114.7, 116.8, 118.4, 119.1, 123.2, 130.4, 132.1, 134.4, 138.3, 144.2 (Ar-C), 123.7 (CF_3_). Mass (ESI, positive mode): 326 (M+H).

Alkali degradant (D1): 2-{[3-(trifluoromethyl)phenyl]amino}benzoic acid, Mol. formula: C_14_H_10_F_3_NO_3_, MW: 281, m. p: 161–163 C UV max: 293 nm, ^1^H-NMR (CDCl_3_, δ ppm): 4.38, 4.81–5.12, 5.4 (8H, cpx, J = 14–15 Hz, Ar-H), 7.22 (1H, s, NH), 10.89 (1H, s, COOH). ^13^C-NMR (CDCl_3_, δ ppm): 113.1, 114.5, 115.9, 118.1, 119.4, 122.3, 130.3, 131.7, 134.0, 139.2, 143.1 (Ar-C), 124.5 (CF_3_), 170.2 (C=O). Mass (ESI, positive mode): 282 (M+H).

## Results and Discussion

### Method Development and Optimization of the Chromatographic Conditions

In preliminary experiments, the drug was subjected to separation by the reversed-phase mode using water and methanol at pH 3.0 using various % aqueous from 10% to 30%. The drug was able to be separated on the chromatogram, but peak shape was not good. It was noted that % aqueous in the mobile phase had shown a drastic effect on the retention time of ETF. Based on the retention time (t_R_), 20% aqueous was optimized to achieve retention of about 7.5 ± 0.1min. In order to increase the theoretical plates, trials were done by varying the pH from 3.0 to 7.0 and a pH of 6.0 was achieved with a phosphate buffer.

Hence, the conditions were optimized on the C_18_ (Agilent, 250 × 4.6 mm, 5 μ) column with UV-PDA detection with the mobile phase consisting of a phosphate buffer (pH-adjusted to 6.0 with OPA) and methanol in the ratio of 20:80% *v/v.* The injection volume was 20μL and the detection was performed at 286nm. The optimized chromatogram is shown in Figure 2. The method has proven specificity and selectivity by separating the degradants of various stress conditions from etofenamate. It was observed that six major degradation products were formed with retention times of 2.8 ± 0.1min (D1), 3.1 ± 0.2min (D2), 4.0 ± 0.1min (D3), 6.1 ± 0.1min (D4), 10.0 ± 0.1min (D5), 11.9 ± 0.3min (D6), respectively. The resolution among all peaks was significant and was found to be more than 2. The % degradation was about 5–30% and the mass balance was between 92–99% and depended on stress. The results are shown in Table 4.

### Analytical Parameters and Validation

The method was validated as per ICH (Q2) guidelines with respect to selectivity, specificity, linearity, accuracy, precision, robustness, limit of detection (LOD), and limit of quantification (LOQ) (9).

#### Specificity

Forced degradation studies of ETF were used to support the specificity and selectivity of the stability-indicating method. The study was employed on the degradation of ETF by sunlight exposure (for 72h), heat (70°C for 72h), acid hydrolysis (0.1N HCl, kept at RT for 3h), base hydrolysis (0.01N NaOH, kept at RT for 3hrs), water hydrolysis (kept at RT for 5 days), and oxidation (0.3% H_2_O_2_, kept at RT for 48h). Degradants were adequately separated from ETF and significant resolution was found among the peaks, thus the specificity and selectivity of the method was proven. The peak purity was found to be more than 99%.

#### Linearity and Range

The linearity of the detector response to the different concentrations of ETF was studied in the range from 20–90 μg/mL at eight different concentrations. Samples were analyzed in triplicate at eight different concentrations, such as 20, 30, 40, 50, 60, 70, 80, and 90μg/mL. The correlation coefficient (r^2^ value), obtained was 0.9992 and indicated a linear response of ETF.

#### Accuracy

Accuracy is performed by recovery studies using the standard addition method at 80, 100, and 120% levels of the sample concentration and is detailed in Table 1. Results of the recovery studies were obtained from five replicate analyses and were found to be between 98.65 and 101.58% (acceptance criteria 98–102%).

#### Precision

The results for the intraday and interday precision studies were obtained from three multiples of the same concentration (40μg/mL) in the linearity range. The % RSD values for the intraday and interday precision were < 1% against the acceptance criteria of less than 2. The method was proven to be sufficiently precise and the results are shown in Table 2.

#### Robustness Test

The robustness of the developed method was determined by analyzing the sample under deliberate changes in method parameters, such as a change in flow rate (±0.1mL), % organic phase (± 2%), pH (± 0.2), and wavelength (± 3nm). The method was robust for all the parameters tested and the % RSD was less than 2%.

#### Limit of Detection and Limit of Quantification

The LOD and LOQ were determined based on the signal-to-noise ratio. The S/N ratio of 3:1 was taken for the LOD and 10:1 for the LOQ. The LOD and LOQ were found to be 0.16 μg/mL and 0.51 μg/mL, respectively. The predicted concentrations of the LOD and LOQ were prepared and injected into the optimized conditions in triplicate (Table 3).

### Forced Degradation

#### Acid-Induced Degradation

Upon treatment with 0.1N HCl at RT for 3h, 24.71% degradation was observed and was optimized for specificity. There was one degradant peak observed at a retention time of 3.1 min (D2). The area percentage of the D2 peak is 19.99% and assay of the ETF was found to be 75.63% as shown in Figure 3. The mass balance of this study was 95.62%.

#### Base-Induced Degradation

When the drug was exposed to 0.01N NaOH, drastic degradation was observed within 1h. The drug degradation was 25.45%, with degradants D1 and D6 at retention times 2.7min and 11.7min with the respective areas of 11.4% and 14.0%, respectively. The mass balance of the study was about 99.99% with an assay value of 74.55% for the ETF chromatogram shown in Fig. 4.

#### Neutral Degradation

A degradation of 10.23% degradation was observed for ETF after 5 days at room temperature. A total of three degradants namely, D2, D3, and D6, were detected at 3.1min, 4.0min, and 11.6min, respectively. The degradation rate was very slow and chromatogram is shown in Fig. 5.

#### Oxidative degradation

Upon treatment of ETF with 0.3% H_2_O_2_ at RT for 48h, 10.12% degradation was observed with one major degradant peak (D6) at 7.2min. The peak area of D6 was 7.23% and the assay value for ETF was found to be 89.88% as shown in Figure 6. In this experiment, the peroxide peak was observed at 2.9 min. In order to predict the degradant D1’s coelution with the peroxide peak, the area of peroxide was monitored. It was concluded that no degradant was formed at 2.1 min.

#### Thermal Degradation

When the was drug exposed to dry heat in an oven at 70°C for 72h, the five degradation products D2, D3, D4, D5, and D6 were detected. Times of 3, 2, 4.0, 6.1, 10.0, and 11.8min showed significant changes in the peak area of the parent drug. The drug degradation was 6.49% with percentage areas of D2, D3, D4, D5, and D6 as 0.68, 0.46, 2.61, 0.51, and 0.27%, respectively. The assay of the active substance in the thermally degraded sample was found to be 93.51% as shown in Figure 7.

#### Photolytic Degradation

The drug was exposed to photolytic degradation under hot sunlight equivalent to 18 times that of 1.2 million lux hours. Degradation of 7.19% was observed with the formation of the three degradants D1, D2, and D6 at retention times of 2.6min, 3.40min, and 11.7min, respectively, as shown in the chromatogram in Figure 8. The percentage degradation of D1, D2, and D6 were found to be 0.35%, 0.44%, and 4.63%, respectively. The assay of ETF was 92.81%.

### Identification of Degradants

In the present stress studies, a total of six degradants were observed, among those three degradants (D1, D2, and D6) were the most common products. D1 (basic) and D2 (acidic) were synthesized and purified by flash chromatography. Although D2 and D6 belonged to base hydrolysis, the yield for D6 was not obtained in the chemical synthesis. It may be due to the instability of D6 under reaction conditions or may be a liquid state product.

The obtained D1 and D2 were characterized by ^1^H-NMR, ^13^C-NMR, and mass spectral data. Retention times of the elucidated degradants for D1 and D2 were 2.7 and 3.1min, respectively. It was confirmed by spike analysis of the isolated D1 and D2 with ETF. D1 was yellow with silky solid crystals, soluble in methanol, sparingly soluble in water, and gave effervescence with sodium carbonate that indicated the presence of a COOH group in the structure. It might be due to the cleavage of the ester linkage in ETF under alkaline conditions. Furthermore, the structure was supported by the presence of a proton signal at 10.9 ppm for OH (carboxyl) and by the absence of the aliphatic proton signal in the NMR spectrum. However, the molecular mass and carbon NMR spectra confirm the structure of the alkali degradant D1 as 2-{[3-(trifluoromethyl)phenyl]amino}benzoic acid.

**Fig. 1:**
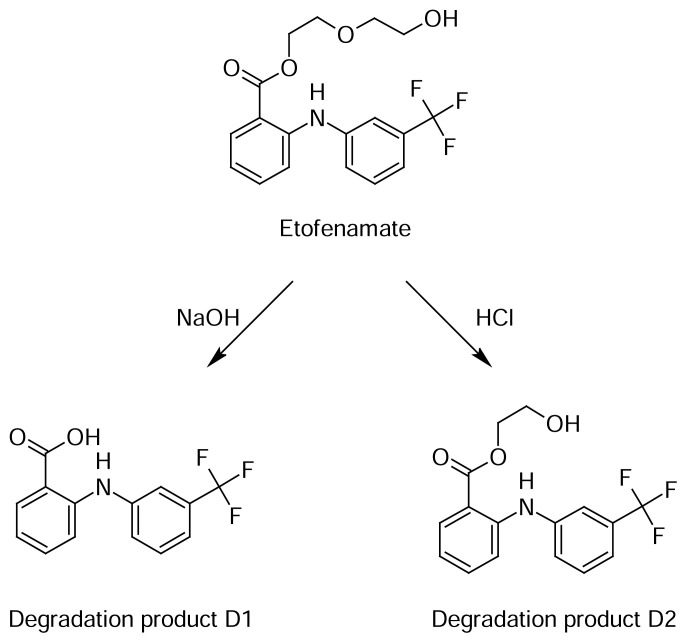
Structure of etofenamate and its identified impurities

**Fig. 2:**
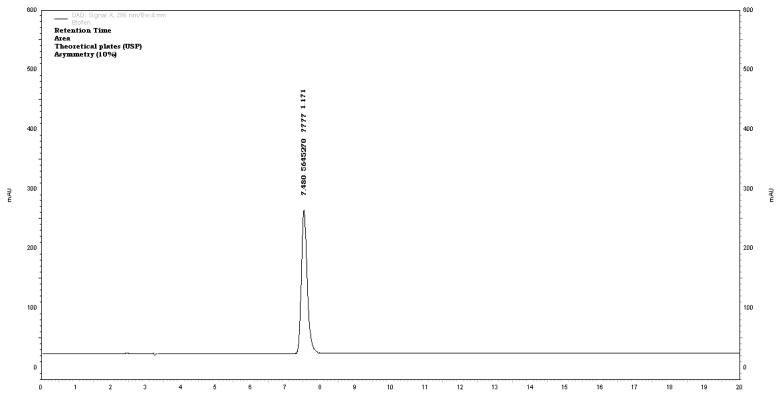
Optimized chromatogram on C18 column

**Fig. 3:**
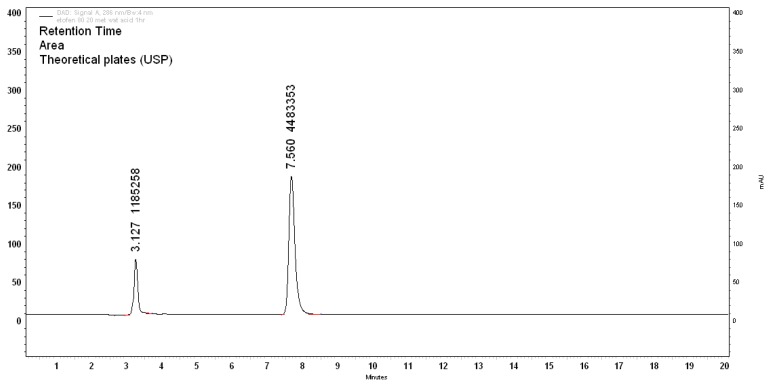
Acid degradation (0.1N HCl, 3h)

**Fig. 4:**
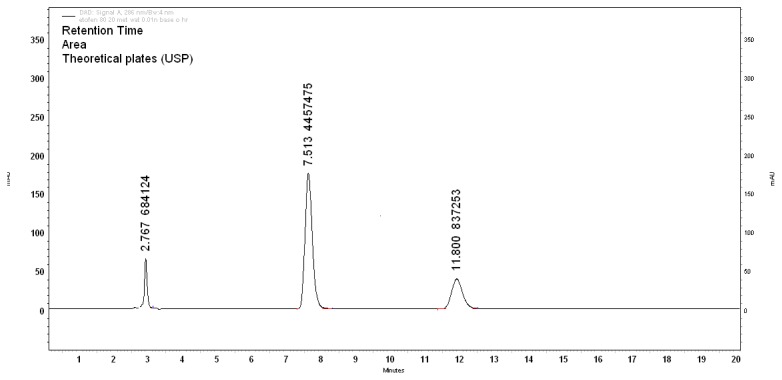
Base degradation (0.01N NaOH, 1h)

**Fig. 5:**
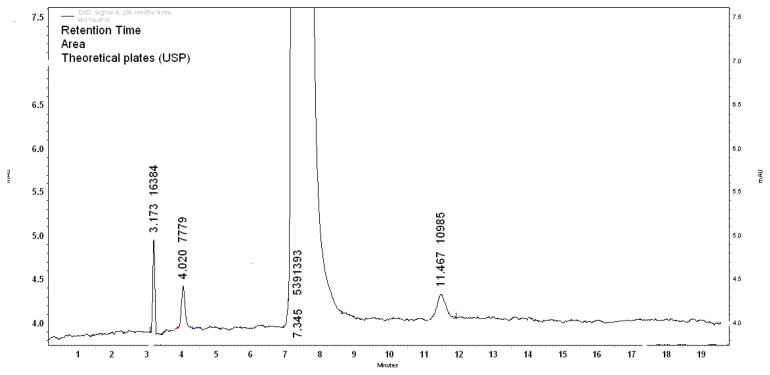
Neutral stress (water, 5 days)

**Fig. 6:**
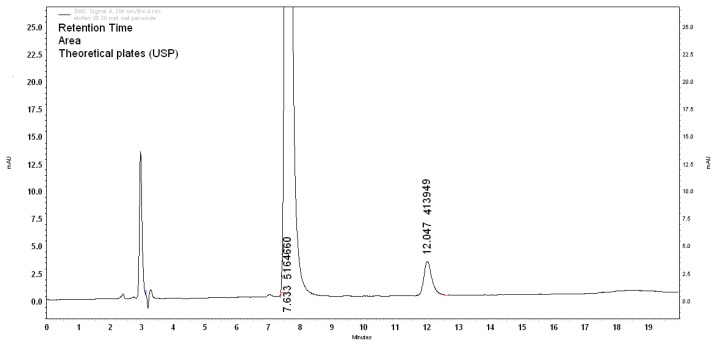
Peroxide stress (0.3% H_2_O_2_, 48h)

**Fig. 7:**
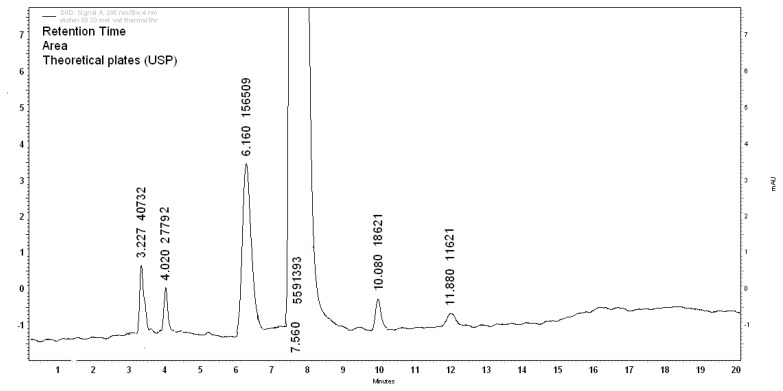
Thermal stress (heat NMT 70 °C, 72h)

**Fig. 8:**
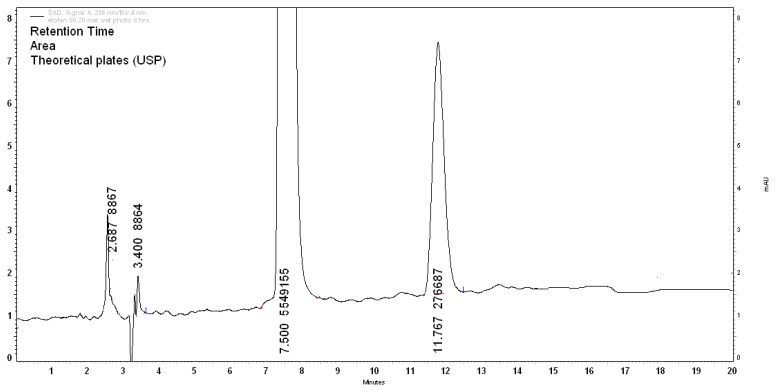
Photolytic stress (hot sunlight, 72h; 4h of sunlight exposure equals to 1.2 million lux hours)

The D2 degradant belonged to acid stress and was detected at 3.1 min. D2 was a pale-yellow viscous liquid, insoluble in water, and soluble in methanol. It was elucidated as 2-hydroxyethyl 2-{[3-(trifluoromethyl)phenyl]amino}benzoate with a molecular weight of 325. The characteristic feature of the structure was confirmed by the loss of two aliphatic carbon signals in the carbon NMR spectrum. Structures of D1 and D2 are shown in Fig. 1.

## Conclusion

A simple, specific, stability-indicating RP-HPLC method was developed for the estimation of etofenamate (ETF) in pharmaceutical dosage form and validated according to ICH guidelines. The method was found to be specific for the detection of all possible impurities in the dosage form under various stress conditions.

## Figures and Tables

**Tab. 1:** Accuracy of the method

Conc. (μg/mL)	Recovery level	Added drug (μg/mL)	Amount recovered (μg/mL)	% Recovery	%RSD (n=5)
40	80%	32	31.57	98.69	0.21
	100%	40	40.63	101.57	0.80
	120%	48	48.41	100.87	1.06

**Tab. 2:** Intra- and Interday Precision

S. No.	Conc. (μg/mL)	Intra-day (n=3) Conc ± SD	Intra-day %RSD	Inter-day (n=3) Conc ± SD	Inter-day %RSD
1	30	30 ± 0.23	0.78	30 ± 0.25	0.84
2	40	40 ± 0.19	0.49	40 ± 0.38	0.97
3	50	50 ± 0.45	0.90	50 ± 0.47	0.95

**Tab. 3:** Validation Parameters

Parameters	Result of the method for ETF
Retention time	7.5 ± 0.1 min
Theoretical Plate	7776 ± 21
Tailing factor	1.17 ± 0.05
LOD	0.16 ± 0.01 μg/mL
LOQ	0.51 ± 0.01 μg/mL
Linearity	20–90 μg/mL
Accuracy	98–101%
Intraday Precision	0.49–0.90 (% RSD)
Interday Precision	0.84–0.97 (% RSD)
Robustness	pH (± 0.2), Flow rate (±0.1 ml), % organic phase (± 0.2%)

**Tab. 4:** Degradation data of Etofenamate (ETF)

Stress Condition	No. of Degradants	% Degradation at t_R_						% Assay of ETF

		2.7	3.15	4.0	6.15	10.10	11.75	
0.1 N HCL (3h)	1	–	19.99	–	–	–	–	75.63
0.01 N NaOH (1h)	2	11.44	–	–	–	–	14.0	74.55
3% H_2_O_2_ (48 h)	1	–	–	–	–	–	7.23	89.88
Heat 70°C (72 h)	5	–	0.68	0.46	2.61	0.47	0.51	93.51
Photolytic (72 h)	3	0.35	0.44	–	–	–	4.63	92.81
Neutral (5 Days)	3	–	0.58	0.42	–	–	0.34	97.98
